# Modulation of Iberian Ribbed Newt Complement Component C3 by Stressors Similar to those Encountered during a Stay Onboard the International Space Station

**DOI:** 10.3390/ijms20071579

**Published:** 2019-03-29

**Authors:** Nathan Guéguinou, Jérémy Jeandel, Sandra Kaminski, Sarah Baatout, Stéphanie Ghislin, Jean-Pol Frippiat

**Affiliations:** 1 Stress Immunity Pathogens Laboratory, EA 7300, Faculty of Medicine, Université de Lorraine, 9 avenue de la Foret de Haye, F-54500 Vandœuvre-lès-Nancy, France; nathan.gueguinou@laposte.net (N.G.); jeremy.jeandel@univ-lorraine.fr (J.J.); sandra.kaminski@univ-lorraine.fr (S.K.); stephanie.ghislin@univ-lorraine.fr (S.G.); 2 Radiobiology Unit, SCK·CEN, Boeretang 200, B-2400 Mol, Belgium; sarah.baatout@sckcen.be

**Keywords:** spaceflight, gravity, amphibian, immunity, inflammation

## Abstract

The complement system plays an important role in inflammation, innate and acquired immunity, as well as homeostasis. Despite these functions, the effects of spaceflight conditions on the complement system have not yet been intensively studied. Consequently, we investigated the effects of five types of chronic stressors, similar to those encountered during a stay onboard the International Space Station, on C3 expression in larvae of the urodele amphibian *Pleurodeles waltl*. We focused on C3 because it is a critical component of this system. These studies were completed by the analysis of adult mice exposed to two models of inflight stressors. Our data show that simulating space radiation, or combining a modification of the circadian rhythm with simulated microgravity, affects the amount of C3 proteins. These results suggest that C3 expression could be modified under real spaceflight conditions, potentially increasing the risk of inflammation and associated tissue damage.

## 1. Introduction

Since Yuri Gagarin became the first human to leave the confines of Earth in 1961, an increasing number of humans have traveled to space and permanently inhabited space stations (Mir and then the International Space Station (ISS)) have been constructed. Studies performed on humans or animals sent to these stations, or subjected to ground-based models used to simulate space conditions, have revealed that these missions induce physiological dysregulations such as muscle atrophy, bone demineralization, cardiovascular and metabolic dysfunctions, impaired cognitive processes, and reduced immunological competence.

Immune system dysregulation occurs during flight and persists during 6-month orbital spaceflight [1,2]. A recent study revealed that about 50% of the astronauts who spent six months onboard the ISS faced immunological problems [3], thereby confirming in-flight dysregulation distinct from the influences of landing and readaptation following deconditioning [1,3,4]. All compartments of the immune system are affected by spaceflight. Concerning innate immunity, it was shown that astronauts’ monocytes exhibit phenotypic and cytokine-production deregulations and a reduced ability to engulf *E. coli*, to elicit an oxidative burst, and to degranulate [5,6,7]. Reactive oxygen species production by macrophages [8] as well as neutrophils phagocytosis and oxidative burst capacities are also significantly reduced [9]. Regarding acquired immunity, several studies reported a reduction of T-cell activation under low gravity conditions [10,11,12]. This phenomenon results from changes in gene expression, cellular interactions, cytoskeleton structure, signal transduction, and disturbed expression of cell cycle regulatory proteins (reviewed in [13,14]). As to humoral immunity, it was shown using the urodele amphibian *Pleurodeles waltl* as animal model [15] that spaceflight affects antibody production in response to an antigenic stimulation [16,17,18]. This was recently confirmed in mice subjected to hindlimb unloading, a model frequently used to simulate the effects of microgravity exposure [19]. Hypergravity and simulated microgravity also impaired the proliferative responses of murine B-lymphocytes [20,21]. Finally, the maturation of immune cells belonging to the myeloid [22,23,24,25,26], B- [27,28,29], and T- [30,31,32] lineages were shown to be reduced under low gravity conditions.

It therefore appears that immunological changes induced by space, an adverse environment in which human and animals face a combination of stressors (e.g., radiation, microgravity, confinement, isolation, and disrupted circadian rhythm), must be seriously investigated and understood to be able to preserve astronauts’ health during future deep-space exploration missions such as the deployment of a Lunar station followed by multiple Mars flyby missions.

In this context, the effects of spaceflight conditions on the complement system, which is key for immune surveillance and homeostasis, need to be studied. Complement constitutes a first line of defense against microbial intruders and orchestrates immunological and inflammatory processes [33]. Indeed, opsonization by complement fragments and pro-inflammatory signaling by anaphylatoxins (resulting from complement activation) recruits macrophages and enables phagocytosis and the formation of a lytic membrane attack complex on cells such as gram-negative bacteria. Complement coordinates innate immunity in cooperation with Toll-like receptors (TLR) [34], links innate response to both humoral and cellular adaptive immunity [35], and regulates the mobilization of hematopoietic stem-progenitor cells from bone marrow to replenish the immune system [36]. It also contributes to the resolution of inflammation by promoting the safe clearance of apoptotic cells and immune complexes [37,38]. Moreover, it participates in homeostasis by promoting tissue repair [39,40], potentiating coagulation to limit the spread of infection [41]; it is also implicated in synaptogenesis (it eliminates weak or immature synapses) [42] and in the differentiation and migration of neural progenitor cells [43]. Finally, it is established that any trigger that tips the delicate balance between complement activation and regulation can induce self-attack and lead to various immune, inflammatory, neurodegenerative, ischemic, and age-related diseases [33].

Given this functional repertoire, and a recent study having shown that hypergravity (an acute stressor faced during takeoff and landing) increases in a g-dependent manner C3 complement component expression [29], we investigated whether chronic stressors encountered during a space mission could affect the expression of this molecule conserved from amphibians to mammals [44,45]. We focused on C3 because it is the most abundant complement protein and all complement activation pathways (extracellularly through the classical, lectins, or alternative pathways or intracellularly via a cathepsin-dependent mechanism) converge at the level of this component [46].

## 2. Results

**Figure 1:**
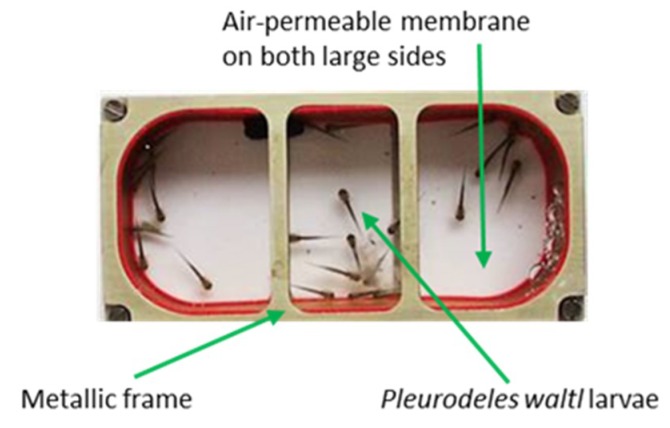
Miniaquarium developed by EADS (European aeronautic defense and space company)-Astrium and the German Space Agency to allow amphibian embryo development onboard the international space station (ISS). These miniaquaria measure 80 mm in length by 40 mm in width and 20 mm in height, and have two transparent sides that are permeable to O_2_ and CO_2_. Once in the ISS, these miniaquaria are placed in the Kubik incubator to maintain temperature at 20 °C.

Different groups of *P. waltl* larvae at the same developmental stage were subjected, in parallel, to various environmental modifications similar to those encountered during a mission onboard the ISS or some combination thereof. These stressors were recreated in the laboratory to the best of our technical abilities and were applied for a maximum of 10 days. Experiments were performed on larvae, and not adults, because many of them can be accommodated in small aquaria (miniaquaria—Figure 1) conceived to allow amphibian embryo development onboard the ISS.

### 2.1. Effects of Simulated Microgravity, Circadian Rhythm Modification, Confinement, Vibration, and Radiation on P. waltl C3 Transcription

To determine how these five individual stressors affect C3 transcription, *P. waltl* larvae were exposed to simulated microgravity or darkness during 10 days, confinement, vibration, or a simulation of space radiation. The total dose of radiation mimicked the one received during a 10-day-stay in the Columbus Laboratory of the ISS [47,48], because miniaquaria were developed to fit within the Kubik incubator located in that part of the ISS. Then, C3 transcripts were quantified by RT-qPCR.

**Figure 2:**
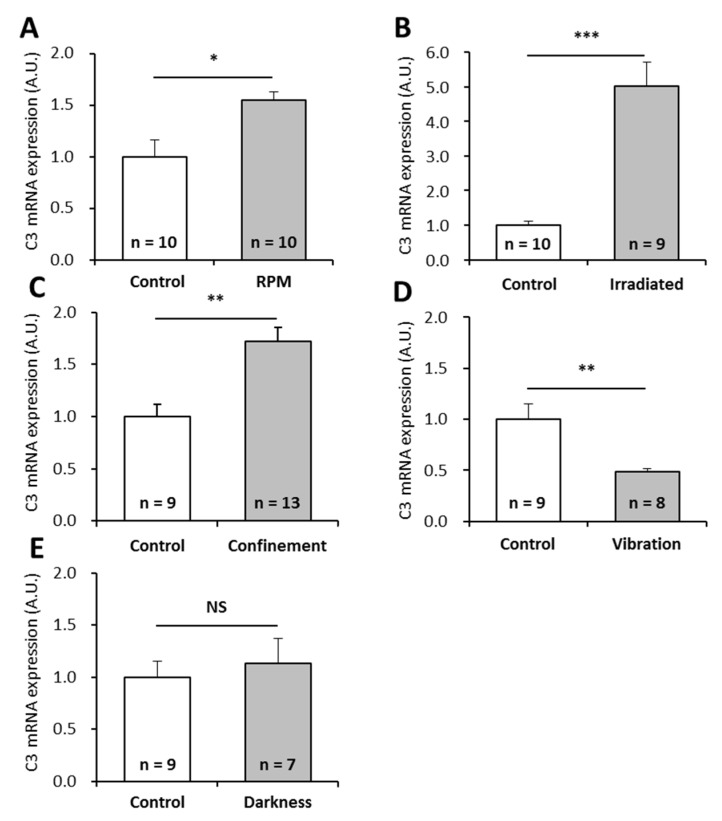
Effects of individual stressors, similar to those encountered during a stay in the ISS, on *P. waltl* complement C3 mRNA levels. Complement C3 mRNAs were quantified by RT-qPCR in larvae subjected to (**A**) simulated microgravity, (**B**) simulated ISS radiation environment, (**C**) confinement, (**D**) vibration, or (**E**) obscurity. Relative values obtained with unstressed controls were set to 1. Results are expressed as means ± SEM. “A.U.” stands for “arbitrary units”. Differences were found using Student’s *t*-tests or Mann—Whitney tests. * *p* < 0.05, ** *p* < 0.01, *** *p* < 0.001, NS., no statistical difference.

Our results show that the amount of C3 mRNAs is 1.5 times higher in larvae exposed to simulated microgravity (10^−2^ to 10^−3^ g) using a random positioning machine (RPM) (Figure 2A), 5 times higher in larvae subjected to the ISS radiation environment (Figure 2B), 1.7 times higher in larvae subjected to confinement (Figure 2C), and 0.5 times lower in larvae subjected to vibration (Figure 2D). The perturbation of the circadian rhythm (darkness as miniaquaria are in the dark within the Kubik incubator) did not affect the amount of C3 mRNAs (Figure 2E). Taken together, these results indicate that C3 expression is impacted at the transcriptional level by numerous spaceflight-associated stressors.

### 2.2. Effects of Two Combinations of Stressors on P. waltl C3 Expression

**Figure 3:**
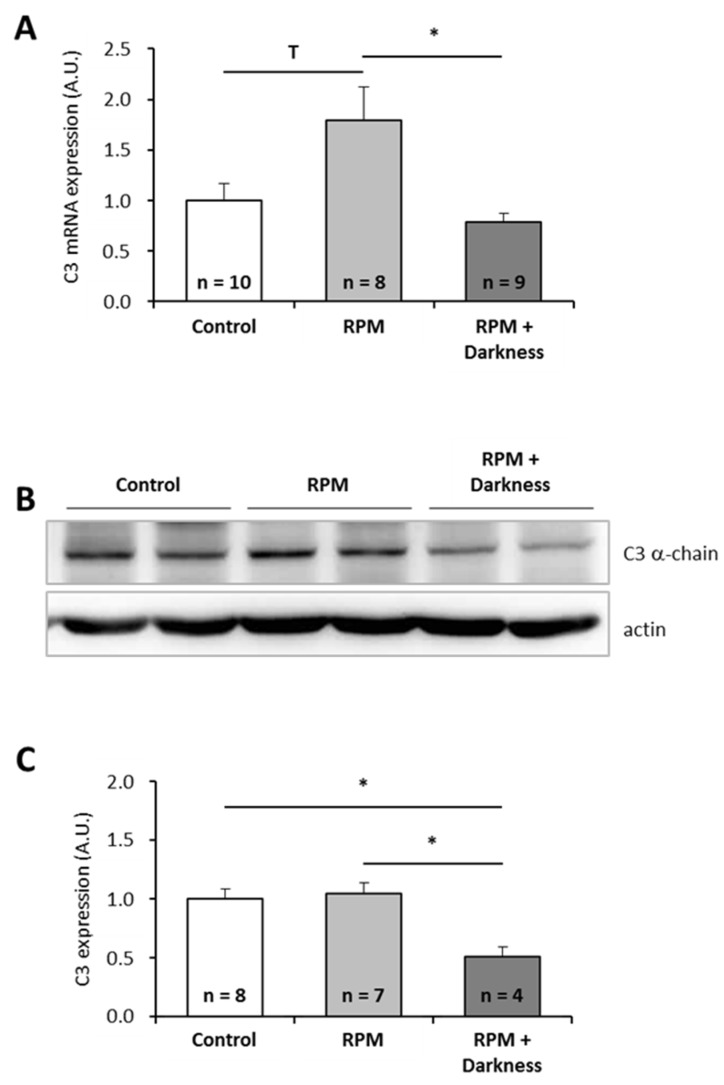
Effects of the combination of simulated microgravity and darkness on *P. waltl* complement C3 expression. (**A**) C3 mRNA were quantified by RT-qPCR. (**B, C**) C3 proteins were quantified by western blotting. Western blot results were visualized by chemiluminescence, analyzed by densitometry, and normalized to α-actin. Relative values obtained with control larvae placed in a miniaquarium positioned on the base of the random positioning machine (RPM) and subjected to normal light conditions were set to one in (**A**) and (**C**). Results are expressed as means ± SEM. “A.U.” stands for “arbitrary units”. Differences were found using Kruskal–Wallis followed by a Dunn test (**A**) or ANOVA followed by a Tukey–Kramer test (**C**). * *p* < 0.05, T indicates a tendency (statistically significant difference with a 10% alpha risk but not with a 5% alpha risk), NS., no statistical difference.

**Figure 4:**
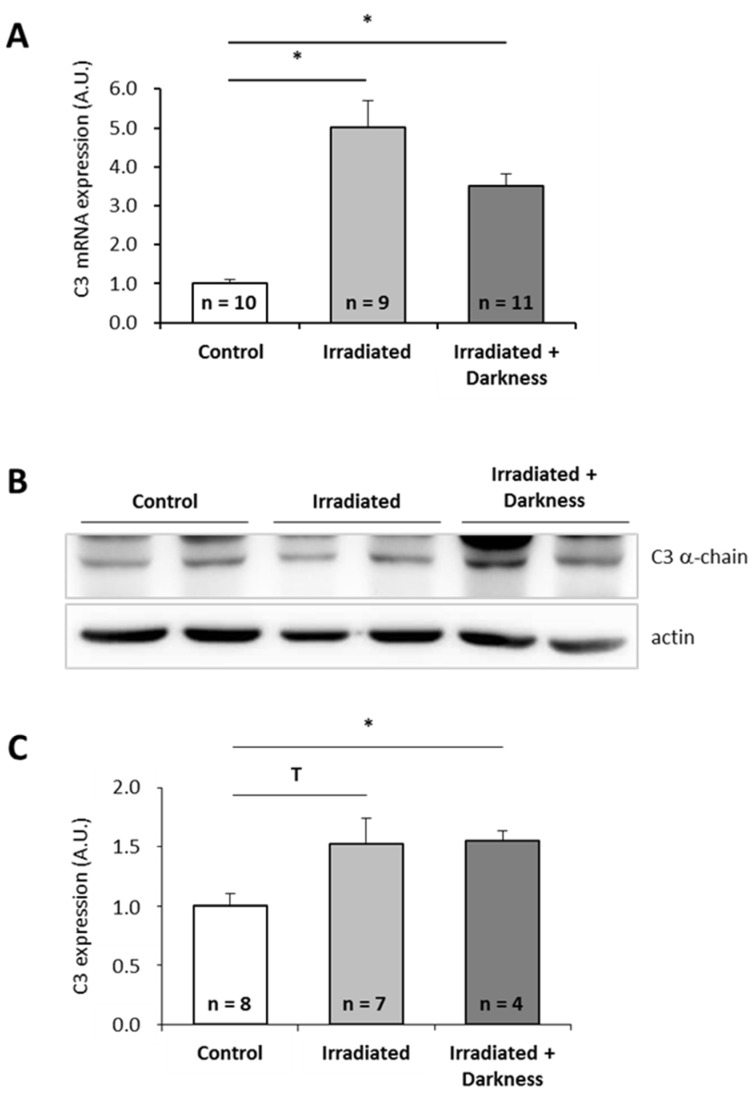
Effects of the combination of radiation and darkness on *P. waltl* complement C3 expression. (**A**) C3 mRNA were quantified by RT-qPCR. (**B, C**) C3 proteins were quantified by western blotting. Western blot results were visualized by chemiluminescence, analyzed by densitometry and normalized to α-actin. Relative values obtained with control larvae reared in a nonirratiated miniaquarium subjected to normal light conditions were set to one in (**A**) and (**C**). Results are expressed as means ± SEM. “A.U.” stands for “arbitrary units”. Differences were found using Kruskal–Wallis followed by a Dunn test. * *p* < 0.05, T indicates a tendency (statistically significant difference with a 10% alpha risk but not with a 5% alpha risk), NS., no statistical difference.

Since astronauts are submitted to a combination of stressors during spaceflight, and not to single stressors as tested above, we wondered how combinations of individual stressors would affect C3 expression. Because it is technically impossible to test all possible combinations, we focused on two combinations of stressors that could be simulated in the laboratory: the combination of simulated microgravity with darkness (Figure 3) and the combination of space radiation with darkness (Figure 4). Interestingly, exposure of larvae to simulated microgravity and darkness did not affect C3 mRNA level (Figure 3A). Thus, it appears that combining simulated microgravity with darkness annuls the effect of microgravity on C3 mRNA expression. However, the amount of C3 proteins was decreased by a factor of two by comparison to controls (Figure 3B,C). Exposure of larvae to simulated space radiation and darkness led to a 3.5-fold increase of C3 mRNA level (Figure 4A) and a 1.5-fold increase of C3 proteins (Figure 4B,C) in comparison to controls. Combining darkness with space radiation did not statistically change the amount of C3 mRNAs and proteins when compared to space radiation alone.

### 2.3. Effects of Spaceflight Stressors on Murine C3 Expression

**Figure 5:**
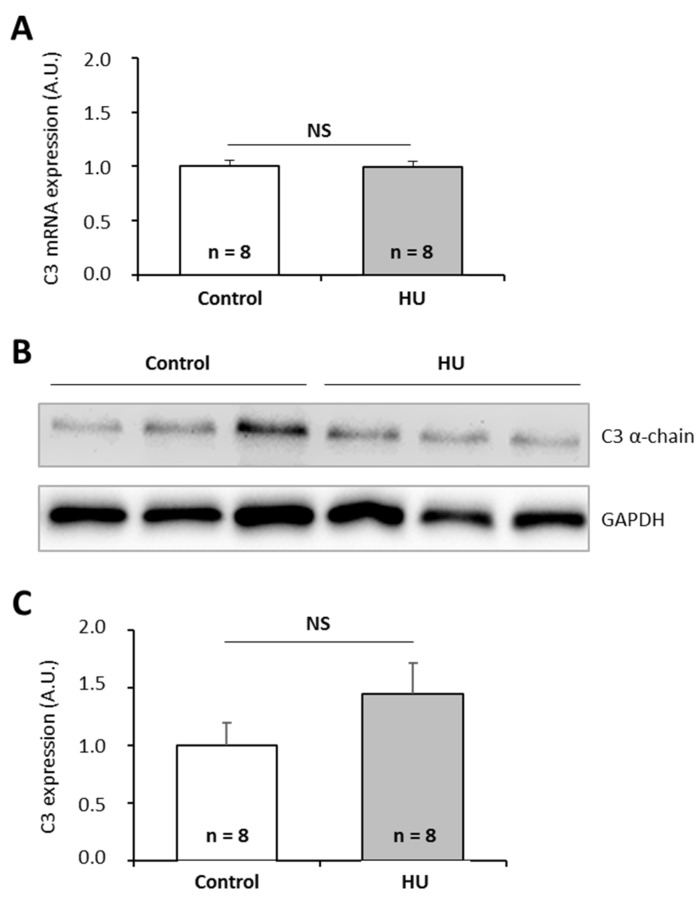
Effects of the hindlimb unloading model, used to simulate microgravity, on murine complement C3 expression. (**A**) C3 mRNAs were quantified by RT-qPCR. (**B, C**) C3 proteins were quantified by western blotting. Western blot results were visualized by chemiluminescence, analyzed by densitometry, and normalized to GAPDH. Relative values obtained with control mice reared under normal housing conditions were set to one in (**A**) and (**C**). Results are expressed as means ± SEM. “A.U.” stands for “arbitrary units”. No differences were found using student’s *t*-tests. NS., no statistical difference.

**Figure 6:**
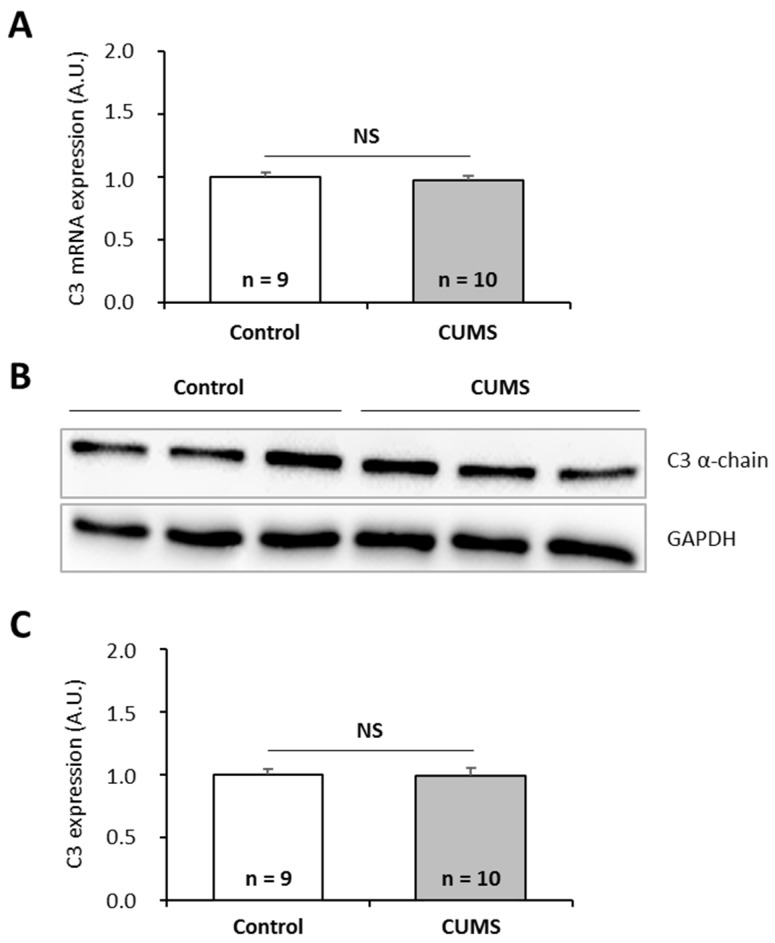
Effects of the CUMS (Chroni Unpredictable Mild psychosocial and environmental Stressors) model, used to simulate socio-environmental stressors encountered during a stay in a space station, on murine complement C3 expression. (**A**) C3 mRNAs were quantified by RT-qPCR. (**B, C**) C3 proteins were quantified by western blotting. Western blot results were visualized by chemiluminescence, analyzed by densitometry, and normalized to GAPDH. Relative values obtained with control mice reared under normal housing conditions were set to one in (**A**) and (**C**). Results are expressed as means ± SEM. “A.U.” stands for “arbitrary units”. No differences were found using student’s *t*-tests. NS., no statistical difference.

To further investigate the effects of chronic spaceflight-associated stressors, we exposed mice to hindlimb unloading (HU) used to simulate microgravity [49] or to the CUMS model used to simulate socio-environmental stressors encountered during a stay in a space station [50]. CUMS implies submitting mice to confinement, isolation, disrupted circadian rhythm, interpersonal issues, perturbation of spatial references, lower dietary intake, and uncomfortable living conditions in a chronic and unpredictable manner. This model replicates some spaceflight-induced immunological changes [50]. Then, we quantified C3 mRNAs and proteins in the liver of these animals because this tissue is the major source of complement. Our data show that HU and CUMS exposures did not affect the expression of C3 at the mRNA and protein levels (Figure 5 and Figure 6), indicating that simulations of chronic socio-environmental stressors or of microgravity did not affect C3 expression. This last observation is in agreement with the fact that RPM exposure did not modify the amount of *P. waltl* C3 proteins (Figure 3C).

## 3. Discussion

During spaceflight, humans and animals are subjected to various environmental modifications. In this study, *P. waltl* larvae were exposed to chronic stressors similar to those encountered during a stay in the ISS (microgravity, perturbed circadian rhythm, radiation, confinement, and vibration) to determine how these individual stressors, but also two combinations of them, affect the expression of a critical component of the complement system. *P. waltl* larvae were used as model organisms because (i) they fulfills many technical requirements associated with spaceflight experimentation; (ii) a hardware has been developed to allow amphibian development onboard the ISS; (iii) this model was shown to be useful for improving our understanding of the immunosuppressive effects of spaceflight [15] and (iv) the cardinal elements of its immune system are conserved [51,52,53], including C3. These studies were completed by the analysis of mice exposed to two models of inflight stressors.

We noted that, in complement of a previous study having shown that hypergravity exposure increases murine C3 protein expression [29], simulating space radiation or combining simulated microgravity with darkness affects the amount of *P. waltl* C3 proteins, thereby suggesting that C3 expression could be modified under real spaceflight conditions. Indeed, it is known that the complement system is sensitive to disturbances of homeostasis or assault [54]. Furthermore, as reported here, previous studies showed (i) that radiotherapy upregulates human and murine C3 at mRNA and protein levels [55], (ii) that head-down tilt bed, used to simulate weightlessness, does not change human plasma complement factor C3 concentration [56], and (iii) that sleep deprivation does not affect circadian fluctuation of human C3 and C4 plasma concentrations [57].

Changes of C3 mRNA and protein levels could be mounted by *P. waltl* larvae to neutralize harmful free radicals induced by stressors. Indeed, previous studies have shown that spaceflight can cause oxidative damage and the generation of reactive oxygen species (ROS) in humans, rodents, and amphibians [58,59,60,61], and that complement plays an important role in the inflammatory process after oxidative stress [62]. Furthermore, there is evidence that ROS activate the complement system and that C3 provides protection against oxidative stress [62,63,64]. These data might explain why Baqai and colleagues noted that exposure to the spaceflight environment increases murine anti-inflammatory mechanisms [65]. Another possibility is that danger signals, such as heat shock proteins (a hallmark of stressed cells and organisms is the increased synthesis of HSPs), whose expression is affected by spaceflight [66], regulate C3 expression, as shown, for example, in photodynamic therapy-treated tumors [67]. This hypothesis is supported by the presence of heat shock binding sites in human, mouse, and rat C3 promotors [45]. Given the conservation of C3 across vertebrates, it is very likely that the promotor of the *P. waltl* C3 gene also contains such binding sites.

Changes in C3 expression could be involved in numerous spaceflight-associated physiological changes because, besides its obvious involvement in eliminating microbes, complement participates in such diverse processes as synapse maturation, clearance of immune complexes, angiogenesis, mobilization of hematopoietic stem-progenitor cells, tissue regeneration, and lipid metabolism [33]. Furthermore, this system is required at early stage of animal development, for example, at *P. waltl* [45] and *X. laevis* [68] neurulation stages but also for the proper migration of neural crest cells during early vertebrate development [69].

In the future, it will be interesting to quantify complement activation products, because numerous factors can induce the production of these molecules. Indeed, in addition to pathway-dependent activations, coagulation factors such as thrombin, plasmin, FXa (factor Xa), and FXIa (factor XIa) have been shown to induce the cleavage of C3 and C5 proteins promoting the generation of C3a and C5a fragments [70]. Furthermore, radiation upregulate C3a and C5a production [55] and modified membranes of late apoptotic and necrotic cells are potent activators of complement [33]. Thus, changes in coagulation cascade protein expression noted during and after spaceflight [29,71,72], space radiation, and apoptosis, which is frequently increased in microgravity [73,74,75,76], could increase the production of C3a and C5a. Since these molecules have pro-inflammatory properties and increase the expression of MHC (major histocompatibility complex) class II and costimulatory molecules on antigen presenting cells such as dendritic cells, they could influence T cell activation and polarization towards a tolerogenic or inflammatory profile [77,78]. In support of this hypothesis, a recent in vivo study in which OT-II cells (CD4^+^ T cells expressing a transgenic TCR specifically recognizing chicken ovalbumin) were transferred into mice, which were stimulated with ovalbumin inflight, showed an alteration of tolerance [79] and an increase of pro-inflammatory cytokines produced by murine splenocytes. This last observation could be mediated via extracellular signal-regulated kinase (ERK) and phosphatidylinositol 3-kinase (PI3K) pathways [80] that are sensitive to microgravity [81,82,83]. Finally, as C3a and C5a anaphylatoxins are powerful chemoattractants for neutrophils, monocytes, and macrophages, the modification of their production could help explain changes in leukocyte distribution noted after space missions [13,14].

In conclusion, the analysis of *P. waltl* larvae submitted to recreated-in-the-lab ISS-associated stressors show that some of them affect C3 expression, potentially increasing the risk of inflammation and associated tissue damages that could affect various organs and physiological systems during future deep-space missions. Future investigations performed on mammals embarked in a space station will be required to confirm this study and to determine if spaceflight affects the production of complement activation products that have numerous essential biological functions. Given the sensitivity of the complement system to a vast array of stimuli, strategies to mitigate the potential increase of complement activation products will likely need to target several factors including the control of microbial load inside the spacecraft and strategies to moderate stress responses such as β-blockers as suggested by Crucian and colleagues [84].

## 4. Materials and Methods

### 4.1. Animals

*P. waltl* embryos at stage 19–20 (3 days after laying) and larvae at stage 34 (11 days after laying) or 36 (13 days after laying) of development, as defined by Shi and Boucaut [85], were used in this study and reared in Evian spring water to avoid potential contamination/infection during treatments.

Three-month-old C57BL/6J male mice were purchased from Janvier Laboratories (Le Genest-Saint-Isle, France). Mice were housed in vented animal cabinets (Noroit, Bouaye, France) under controlled temperature (22 °C) and 12 h light/dark cycles. Prior to the start of the experiments, mice were allowed to rest for one week in groups of four in standard cages.

Animals were treated in accordance with the French Legislation and the Council Directive of the European Communities on the Protection of Animals Used for Experimental and Other Scientific Purposes (2010/63/UE). Local ethic committee approved the protocols (“Comité Ethique Lorrain en Matière d’Expérimentation Animale”, authorizations 04112004, and 20120008).

### 4.2. Simulation of Microgravity Using the RPM

Stage 19–20 embryos were placed in a miniaquarium (Figure 1) developed by EADS-Astrium and the German Space Agency to allow amphibian embryo development onboard the ISS. This miniaquarium was mounted on a desktop random positioning machine (RPM) (Dutch Space, Leiden, Netherlands) placed at 20 ± 2 °C. RPM is a 3D-clinostat classically used to simulate microgravity. Herranz et al. [86] indicated that RPM might be used with very early larval stages (before they can swim freely). This is the case here because, due to its slow development, *P. waltl* hatching occurs 13 days after laying [85], just at the end of RPM exposure. Random speed, direction, and interval, with an angular velocity of rotation between 55 and 65 degrees per second, were applied on the RPM for 10 days. The continuous three-dimensional movement of the samples provided by the RPM randomized the direction of the gravity force, resulting in an average net force approaching zero, therefore simulating microgravity. As negative control, another miniaquarium was placed on the base of the RPM.

### 4.3. Circadian Rhythm Perturbation

Because miniaquaria are kept in the dark in the ISS (they are stored inside the Kubik incubator (Comat Aerospace, Toulouse, France) located inside the Columbus Laboratory), we investigated the effects of continuous darkness. For that purpose, a miniaquarium containing embryos at stage 19–20 of development was kept in the dark 24h/24h during 10 days at 20 °C. A miniaquarium containing embryos at the same developmental stage was subjected for the same duration to natural light/dark cycles for comparison.

### 4.4. Simulation of Space Radiation

The ISS radiation environment was simulated using ^137^Cs γ (low-linear energy transfer (LET)) and ^252^Cf neutron (high-LET particles) sources. ^137^Cs is a mono-energetic source of 0.662 MeV γ rays (LET up to 10 keV μm^−1^). The neutron source ^252^Cf is expelling a neutron spectrum with an average energy of 2.1 MeV (LET up to 250 keV μm^−1^) [87]. Stage 34 *P. waltl* larvae were housed in a miniaquarium and exposed to ionizing radiation under rotation at 2 r.p.m for 65 h within the calibration room facility of SCK•CEN, as previously described [27]. The total dose received during 65 h (30 µSv/h totaling 1.950 mSv, energy of 660 keV of γ rays and 31.2 µ Sv/h totaling 2.028mSv, mean energy of 2.1 MeV of neutrons) imitated the one received during a stay of 10 days in the ISS as determined by Berger et al. [47,48]. One control miniaquarium was kept outside the irradiation bunker at 20 °C for the same duration.

### 4.5. Confinement

*P. waltl* larvae at stage 36 of development were placed in 0.5 mL Eppendorf tubes (one larvae/tube) in 400 µL of water at 20 °C during 7 h to induce confinement. Note that the volume of water could not be lowered and the duration of this experiment could not be extended due to oxygen consumption. Larvae reared under classical conditions were used as controls.

### 4.6. Vibration

To evaluate the impact of vibration, *P. waltl* larvae at stage 36 of development were placed in a Falcon tube containing 50 mL of water at 20 °C and subjected to vibration at 15 Hz for 5 h. Another group of *P. waltl* larvae at the same developmental stage was placed in a Falcon tube and kept at 20 °C on the bench for comparison.

### 4.7. Combinations of Stressors

During a stay in the ISS, animals are simultaneously subjected to the stressors presented above. However, in a laboratory, it is technically impossible to combine all these stressors. Consequently, we focused on two combinations of stressors: (i) the combination of simulated microgravity with darkness and (ii) the combination of simulated radiation with darkness. In the first case, a miniaquarium containing *P. waltl* embryos at stage 19–20 of development was kept in the dark and subjected to simulated microgravity (RPM) during 10 days at 20 °C. Control embryos were placed in a miniaquarium located on the base of the RPM and subjected to normal light conditions. In the second case, a miniaquarium containing *P. waltl* embryos at stage 19–20 of development was kept in the dark during 10 days at 20 °C and subjected to simulated space radiation during the last 65 h. Embryos of the same developmental stage placed in a miniaquarium and reared under standard conditions were used as controls.

### 4.8. Simulation of Microgravity using the Hindlimb Unloading Model

Mice were isolated five days before the beginning of the procedure in standard or hindlimb unloading (HU) cages (312 mm in length, 197 mm in width, and 260 mm in height). Mice were suspended by using a dressing retention sheet wrapped around the tail and a wire hooked on a swivel pulley system as previously described [20,28]. During suspension, mice were provided with food and water ad libitum. Unsuspended control mice were kept individually in same size cages.

### 4.9. Simulation of Socio-Environmental Stressors Encountered During a Space Mission Using the CUMS Model

Mice were isolated and subjected to six mild environmental or psychosocial stressors: 30° cage tilt for 1 h, 2 h, or 15 h; confinement in a small cage (110 × 80 × 80 mm) for 1 h or 2 h; paired housing for 2 h; one 15 h overnight period with difficult access to food without a reduction in the daily food ration; one 15 h overnight period with permanent light; and one 15 h overnight period in a soiled cage (50 mL of water in 1000 mL of bedding) as previously described [50,88]. Control mice were housed by five in standard cages placed in another room of the animal facility.

### 4.10. RT-qPCR

At the end of treatments, *P. waltl* larvae and mice liver were homogenized in TRIzol reagent (Invitrogen, Carlsbad, CA, USA) for RNA extraction according to the instructions of the manufacturer. RNA was reverse transcribed using random primers, RNAout, and MML-V (Moloney murine leukemia virus) reverse transcriptase (Invitrogen, Cergy Pontoise, France). qPCR were then performed. Primers specific for *P. waltl* and murine C3 as well as housekeeping transcripts (Table 1) were designed using the Genscript software (http://genscript.com/ssl-bin/app/primer) in different exons to ensure that they could not hybridize to potential traces of genomic DNA. The specificity of each primer pair was checked with BLAST (basic local alignment search tool, NCBI, Bethesda, MD, USA). The Mesa Green qPCR Master Mix (Eurogentec, Seraing, Belgium) and a Mastercycler^®^ realplex^2^ real-time PCR machine (Eppendorf, Hamburg, Germany) were used to performed real-time PCR. We used the following cycling protocol: 3 min at 95 °C, 40 cycles of 15 s at 94 °C, and 30 s at the annealing temperature indicated in Table 1. Amplification efficiencies were controlled with standard curves and validated in the range of 90%–110%. Each qPCR was performed in triplicate. Data were analyzed using the Pfaffl model [89]. Relative expressions, expressed in arbitrary units (A.U.), were calculated by comparison to housekeeping transcripts using GeNorm software and Vandesompele’s methodology [90].

### 4.11. Western Blotting

Proteins were prepared by lysing *P. waltl* larvae in lysis buffer containing 50 mM HEPES pH 7.4, 150 mM NaCl, 2 mM EDTA, 1% Triton X-100, 0.1% SDS, 10% glycerol, 50 mM NaF, and 1 mM Na_3_VO_4_ or 20–25 mg of mice liver in lysis buffer containing 15 mM Tris pH 7.4, 150 mM NaCl, 1 mM EDTA, and 1% Triton X-100. Both buffers were supplemented with proteases inhibitor. Forty µg of proteins from *P. waltl* larvae and 30 µg of proteins from mice liver were heated at 95 °C for 5 min, run on 8% SDS-polyacrylamide gels, and electrotransferred to PVDF (polyvinylidene difluoride) membranes (Amersham, Buckinghamshire, UK). Membranes were incubated with antibodies detecting human and murine complement C3 alpha chain (GTX101316, Tebubio, Le Perray-en-Yvelines, France). As analysis revealed several bands, the specific one was identified based on its molecular weight and quantified. Then, membranes were stripped (stripping buffer from Thermo Fisher Scientific, Waltham, Massachusetts, USA) and incubated with an antibody against α-actin for *P. waltl* samples or an antibody against GAPDH for murine samples. Immunodetection was performed using the Pierce ECL western blotting substrate (Thermo Fisher Scientific, Illkrich, France). Signals were visualized by chemiluminescence (FX7, Vilbert-Lourmat, Marne la Vallée, France), analyzed by densitometry (ImageJ^®^, NIH, USA), and normalized to α-actin or GAPDH used as housekeeping proteins.

### 4.12. Statistics

Statistical analyses were performed using the StatView software (SAS Campus Drive Cary, NC, USA) and the Anastats website (http://www.anastats.fr/outils.php). Outlier values were determined by a boxplot of each studied group. When normality and homogeneity of variances were ascertained, as determined by Kolmogorov-Smirnov and Fisher tests, respectively, student’s *t*-tests were used to perform two groups’ comparisons. When data were not normally distributed or if there was a heterogeneity of variances, Mann—Whitney tests were used. For three groups’ comparisons, homogeneity of variances was checked using the Levene test. If there was a heterogeneity of variances, Kruskal–Wallis tests were performed followed by a Dunn test for two by two comparisons. In the other cases, ANOVA tests were done followed by a Tukey–Kramer test. Stars indicate statistically significant differences. T indicates trends determined using a 10% alpha risk. All results are shown as means ± SEM.

## Figures and Tables

**Table 1:** Primers used in this study. F, forward. R, reverse.

Target	Sequences	Annealing Temperature (°C)
*P. waltl* C3	F: 5′-TGGTGACAATGACACTGCCT-3′	62
	R: 5′-CATCCACCCAGATGGAGTCT-3′	
Murine *C3*	F: 5′-AGAGGCAAGTGCTGACCAGT-3′	62
	R: 5′-ACTGGCTGGAATCTTGATGG-3′	
*P. waltl* actin	F: 5′-TGGTCGTGACCTGACTGATT-3′	60
	R: 5′-TCACGGACAATCTCACGTTC-3′	
*P. waltl* TAFII	F: 5′-TTCACGAGCTGTCTGTGGAG-3′	60
	R: 5′-CCTGGGAAGCATTTGGTAGA-3′	
*P. waltl* mtRNA16S	F: 5′-CGTGCAGAAGCGGAGATAA-3′	60
	R: 5′-TGTCGGGCTGTTGTAGGG-3′	
*P. waltl* GAPDH	F: 5′-GAAGGTAGTAAGCAACGCCTCCT-3′	65
	R: 5′-CACAGCATGTACAGTGGTCATCA-3′	
Murine Ppia	F: 5′-GTCTCCTTCGAGCTGTTTGC-3′	58
	R: 5′-GCGTGTAAAGTCACCACCCT-3′	
Murine Eef2	F: 5′-GTGGTGGACTGTGTGTCTGG-3′	58
	R: 5′-CGCTGGAAGGTCTGGTAGAG-3′	
Murine Rpl13	F: 5′-GGAAGCGGATGAATACCAAC-3′	61
	R: 5′-CTTGTCATAGGGTGGAGCGA-3′	
Murine Eif3f	F: 5′-CATCAAGGCCTATGTCAGCA-3′	61
	R: 5′-AGGTCAACTCCAATGCGTTC-3′	

## Abbreviations

A.U.	Arbitrary unit
BLAST	Basic local alignment search tool
EADS	European aeronautic defense and space company
ERK	Extracellular signal-regulated kinase
FXa	Factor Xa
FXIa	Factor XIa
HU	Hindlimb unloading
ISS	International space station
MHC	Major histocompatibility complex
MML-V	Moloney murine leukemia virus
PI3K	Phosphatidylinositol 3-kinase
PVDF	Polyvinylidene difluoride
RT-qPCR	Reverse transcription-quantitative PCR
RPM	Random positioning machine
ROS	Reactive oxygen species
TLR	Toll-like receptors
